# Cultural competence in a context of ethnic tension

**DOI:** 10.1186/s13584-019-0317-5

**Published:** 2019-06-07

**Authors:** Eliezer Be’eri, Maurit Beeri, Tal Cohen

**Affiliations:** ALYN Hospital Pediatric and Adolescent Rehabilitation Center, Jerusalem, Israel

**Keywords:** Cultural competence, Medical translation, Israel healthcare system

## Abstract

This commentary describes how the first program for enhancing cultural competence in an Israeli healthcare facility was implemented at the ALYN Hospital Pediatric and Adolescent Rehabilitation Center in Jerusalem, and the lessons that can be learned from the ALYN experience for other healthcare facilities attempting to enhance their cultural competence, particularly in environments of heightened inter-cultural tension. A structured program was developed to educate hospital staff and optimize the hospital’s administrative functioning towards the goal of enhanced cultural competence. The program was initiated with an international conference on site to promote awareness of the concept, and included, among other steps, the appointment of a senior administration “Coordinator of Cultural Competence”, improvements in translation services, regular educational seminars, the opening of a Muslim prayer room in the hospital, and accommodations for Sabbath and Ramadan observance. Enhancing cultural competence was found to be an ongoing work-in-progress, with unanticipated cultural challenges constantly emerging, and demanding ad-hoc solutions. Some elements of the program encountered resistance from members of staff, and occasionally from members of the hospital’s dominant patient cultures. Overall, enhanced cultural competence at ALYN brought benefit to both patients and the institution, ranging from a more pleasant patient experience to improved patient adherence to treatment plans, better patient-caregiver communication and a more positive and cohesive professional team and work environment.

“Cultural competence” refers to the ability of healthcare providers to deliver healthcare services in a manner that is sensitive to the patient’s cultural background [1]. Schuster, Elroy and Rosen [2] recently reported in this journal that, on average, the cultural competence of Israeli hospitals is “low to moderate” in terms of local Ministry of Health (MOH) and international standards, a finding that emphasizes the need to actively promote cultural competence as a value in the Israeli healthcare system. The first hospital in Israel to adopt enhanced cultural competence as an institutional goal was the ALYN Hospital Pediatric and Adolescent Rehabilitation Center (ALYN) in Jerusalem. ALYN is a 120-bed facility for children and young adults with chronic disabilities, providing rehabilitation services for 500 hospitalized in-patients and 50,000 clinic visits per year. The hospital is an independent non-profit organization, unaffiliated with the Israeli health funds or MOH, but under supervision of the MOH and funded by payments from the health funds on a fee-for-service basis. This commentary describes the process that took place at ALYN, and the lessons that other Israeli hospitals can learn from the successes and failures of ALYN’s cultural competence program.

In Western countries, “cultural competence” has developed as a professional value within the broader context of “multiculturalism” as a societal value, possibly because an ethos of respect and tolerance of the “other” characterizes both concepts. In the Middle East, however, ethnic conflict between competing cultures is part of day-to-day life. In situations of ethnic conflict, societies tend to focus on protecting their cultures from the “other”, rather than embracing multiculturalism in the public domain. It is all the more surprising, therefore, that a hospital in Jerusalem, a city at the very heart of the region’s geopolitical conflicts, successfully pioneered the introduction of cultural competence as a value in the Israeli healthcare system.

Motivated by a desire to improve the patient experience in our hospital and to enhance patient adherance with treatment plans, ALYN adopted “improved cultural competence” as an institutional priority in 2007. ALYN first learned about the concept from the Jerusalem Intercultural Center, which accompanied the hospital in devising a program of structured steps and goals designed to educate the hospital’s staff about cultural competence and to revamp the hospital’s administrative structure and functioning in this area. We decided to focus initially on cultural issues related to the Muslim Arab and Jewish Ultra-Orthodox populations, as these were the distinct cultural sub-groups most frequently encountered at ALYN.

The first steps focused on improving the hospital’s cultural competence at the clinical level. A day-long, international conference on “Cultural Competence in Healthcare,” was held in the hospital, so as to raise awareness of the issue and demonstrate ALYN’s commitment to this new institutional objective. Immediately thereafter, a questionnaire was administered to map existing staff attitudes towards, and awareness of, cultural issues in the hospital’s day-to-day functioning. Administered throughout the hospital, the questionnaire had an additional, undeclared, goal: to raise staff awareness of the concept of “cultural competence” and its related ideas and vocabulary, and jumpstart a process of introspection about the topic. The questionnaire was followed by a series of educational seminars for each department, which included a professional actor role-playing cross-cultural issues typically encountered by the hospital’s professional teams in their day-to-day work. These simulations promoted insight into the issues, and enabled the teams to develop cross-cultural problem-solving strategies.

Along with changes at the clinical level, steps were implemented at the managerial level, such that the evolution in ALYN’s organizational culture was simultaneously “bottom-up” and “top-down”. The managerial changes included:

1. A senior-leadership staff position of “Coordinator of Cultural Competence” was created, with responsibility for in-service training programs related to cultural competence and for solving culture-related issues in the hospital. This was a part-time position accounting for approximately 20% of that person’s work hours.
2. A room was dedicated as a Muslim prayer room, in parallel to the existing synagogue. It was the first designated Muslim prayer room in an Israeli hospital serving a mixed Jewish-Muslim population.
3. Sixteen members of staff received training in medical translation between Hebrew and Arabic, Amharic, Russian, or Spanish.
4. The use of Arabic was made equivalent to that of Hebrew in all hospital functioning. This entailed:
  - Reissuing all signage in both Hebrew and Arabic (and where relevant, in English and Russian as well). This included direction signs, room titles, staff name tags, public notices, and the hospital’s vision statement.
  - Translating all hospital documents intended for patient use from Hebrew to Arabic, and printing them in either one or both languages, depending on whether they were intended for use by a specific patient, or intended for general use. These documents included consent forms, daily schedules, receipts, explanations, etc.
  - Translating the hospital’s website, which previously had versions in Hebrew and English, into Arabic.
5. The intake process for orienting new employees prior to their start of work at ALYN was expanded to include a mandatory, one-day, training seminar in cultural competence.

The transition to a more culturally competent institution was not a binary event. Once we implemented the abovementioned changes, we found that the need for new culturally competent solutions never ended. Our increased cultural awareness led to the discovery of new issues that had always existed in our daily work but were previously unnoticed or ignored. Now that they came into focus, they demanded solutions.

In particular, “calendar awareness” emerged as an ongoing challenge. We discovered, for example, that being in the hospital over the Jewish Sabbath was particularly difficult for Orthodox Jewish patients and their families. The hospital therefore made “Shabbat kits” containing candles, grape juice, and other ritual objects available to this population each week, and accommodations were made to enable families to prepare Shabbat food and avoid desecrating the Sabbath within the hospital. Similarly, for the Arab Muslim population, treatment schedules were adjusted in order to accommodate fast times during the month of Ramadan. Festival “fact sheets” explaining customs and traditions were also created and distributed to the hospital staff prior to the major holidays of all religious denominations.

We found spiritual and cultural leaders from outside the hospital to be invaluable advisors on difficult intercultural issues. A rabbinic advisor helped us proactively identify issues that could be problematic for religious Jewish families, helped develop solutions on an ongoing basis, and provided rabbinic guidance for patients on issues of medical “Halacha” (Jewish religious law). We also learned that a patient’s cultural context includes more than just religion; the patient’s understanding of the nature and significance of “illness and disability” and expectations of the form and outcome of “treatment” are also elements of his or her cultural background. If hospital staff address these aspects of patient care in a manner that is discordant with a patient’s cultural assumptions, the stage can be set for misunderstandings, mismatched expectations of treatment, and frustration for both the patients and their families.

When we initiated the program, we expected that improved cultural competence would yield two specific benefits: an improved hospital experience for the patient, and better patient adherence to treatment plans. As the program developed, however, additional, unanticipated, advantages emerged at both the tactical and the strategic levels of hospital functioning. For example, when trying to manage problematic parental or patient behaviors, hospital staff found that cultural factors often played a role; improved cultural competence enabled the staff to understand these situations more clearly and manage them more effectively at the tactical level. Similarly, at the strategic planning level, we found consideration of cultural competence issues to be essential when planning new initiatives and developing annual plans, at all administrative and clinical levels.

We also discovered that having an environment that validated different cultural needs was appreciated not only by patients, but also by members of staff. We were surprised, for example, at the number of Muslim staff members who started using the new prayer room regularly, and were shocked that such a widespread and basic need had gone unnoticed by the hospital administration, and unrequested by the Muslim staff, for many years. Similarly, staff participation in traditional hospital ceremonies such as the annual Rosh Hashanah toast improved markedly once the hospital administration realized that the presence of ceremonial alcohol (or even grape juice) at these events had been making attendance uncomfortable for Muslim workers for years. Replacing the alcohol with fruit or pastries, as a statement of cultural respect, enabled the hospital’s many non-Jewish workers to attend what has now become a unifying, hospital-wide event, that just so happens to be linked to the Jewish New Year.

Not everything was plain sailing. Some cultural competence initiatives met with staff resistance. Dress codes designed to respect the more stringent standards of modesty of traditional Muslim and ultra-Orthodox Jewish patients alienated therapists from more secular cultural backgrounds, who experienced these rules as a cultural imposition. Members of staff, including some senior leaders, had to relearn longstanding, culturally ingrained but not culturally sensitive, ways of addressing people. Those who were incapable of internalizing the new institutional approach were not allowed to continue working at ALYN.

But perhaps the greatest difficulty encountered in optimizing the cultural competency of our patient services was budgetary – specifically, the high cost of adding new staff positions (specifically, for dedicated medical translators) to an already overstretched budget. ALYN dealt with this limiting factor by training existing staff members in the art of medical translation, instead of creating new staff positions.

We also found that improved cultural competence does not necessarily eliminate inter-cultural friction. By its nature, a cultural competence enhancement program focuses on non-dominant sub-cultures. This can prompt a backlash from members of the more dominant culture, particularly in the Middle-East, where public expressions of national culture are often a zero-sum game, in which rival cultures compete for control of the public domain. For example, even though Hanukah candles were lit prominently in the hospital during each night of the holiday, some Jewish families objected to the presence of a Christmas tree in the hospital at the same time, experiencing it as a cultural threat.

Even more problematic were the spikes in inter-cultural tensions that occurred at times of regional geopolitical conflict. During the military clash between Israel and Gaza in 2014, there was the potential for inter-cultural explosions between families watching Israeli and Arab news sources on television, which had widely divergent depictions of the clash between Israel and the Palestinians. This eventually necessitated removing all news channels – Arabic, English, Hebrew and Russian – from the hospital’s TV menu. They have not been reinstated since.

Political tensions were also periodically heightened between staff members, even longstanding colleagues in successful multicultural teams. An institutional ethic valuing cultural competence created an expectation among workers that they, too, would be treated with cultural sensitivity. As a result, during the summer when three Jewish teens and one Palestinian youth were murdered, each by members of the other community, colleagues from the culture of the victim expected expressions of empathy from their workmates of the “other” culture, and when these were not forthcoming (in both directions), tensions spiked. Sensitivities were particularly heightened in this case because relatives of two of the victims, Jewish and Arab, worked at ALYN. We found this conflict of identity politics in the workplace to be unresolvable. Neither open discussion of the issues, nor demanding that workers “leave all politics outside of ALYN”, succeeded in defusing “the elephant in the ward.” Beyond requesting workers to leave their personal points of view on the geo-political situation in Jerusalem outside of in-hospital conversations, our only way forward was to recognize the legitimacy of discomfort on all sides, and acknowledge that neither discussing the issue, nor ignoring it, could resolve it.

In 2011, 4 years after ALYN started its cultural competence program, the Director General of the Israeli MOH issued a directive mandating the development of cultural competence in all Israeli healthcare facilities [3, 4]. Two years after publication of the directive, however, as reported by Shuster et al., cultural competence programs in Israeli hospitals generally remained limited in scope and implementation [2].

What factors might explain this disappointing rate of adoption?

Schuster et al. found that those elements of the directive that had been defined as “mandatory” (such as translating documents, adapting the physical environment, and developing an organizational cultural competence policy) had been better adopted by healthcare institutions than those elements that had been labelled as “recommendations” (such as ongoing staff training, providing oral translation services, and appointing a Director of Cultural Competence). The ALYN experience, however, would suggest that the “mandatory versus recommended” designation per se might not be the primary reason for this difference, because in practice ALYN prioritized implementation of the different elements of its cultural competence program in a manner that paralleled Shuster’s findings for the Israeli healthcare system as a whole, even though the ALYN program predated the MOH directive by several years, before any “mandatory” or “recommended” designations existed. For us, the reason was primarily budgetary: A once-off expense or effort (such as translating a database of documents, or making structural changes to a building) is easier to fund than is an expense that is ongoing (such as adding new staff positions for translation services or for managing a cultural competence program, or for ongoing in-service education programs). This highlights the harsh reality of administering quality healthcare in a financially strapped environment, particularly when new guidelines are issued without provision of the additional budgetary sources needed to implement them. In such circumstances, “soft” programs such as enhancing cultural competence are likely to receive low priority, unless specific cultural competence goals are monitored by the MOH and incorporated as a mandatory element of the criteria for hospital licensing renewal by the MOH.

Another possibility suggested by Schuster is that small size (less than 400 beds), geographic location in the north of the country or Jerusalem (rather than the south or center of the country), or private (rather than government) ownership of a hospital might make it harder for institutions to improve their cultural competence. The ALYN experience, however, would suggest that none of these characteristics, per se, are limiting factors, as all of them are true of ALYN. Indeed, Schuster and colleagues found that immutable hospital characteristics (such as location, ownership or size) could not be independently correlated with degree of cultural competence on multivariate analysis of their findings. In other words, as mentioned by Shuster, all Israeli healthcare facilities are likely to have the ability to substantially enhance their cultural competence.

Every healthcare facility faces its own unique intercultural challenges, and cultural competence enhancement programs must be customized accordingly. By their nature, such programs are a never-ending “work in progress”, and healthcare institutions should approach this undertaking with that in mind. A helpful resource in this regard is an on-line support system (https://groups.google.com/forum/#!forum/cc-health-il) established by the Jerusalem Intercultural Center, which enables hospital cultural competence coordinators to seek advice and share knowledge with local colleagues on an ongoing basis.

We believe that enhanced cultural competence brings benefit to both patients and the institution, ranging from a more pleasant patient experience to improved patient adherence to treatment plans, better patient-caregiver communication and a more positive and cohesive professional team and work environment. These are outcomes that are difficult to quantify. However, our experience at ALYN has been that the efforts to enhance our cultural competence are recognized and appreciated by our patients and their families. We frequently receive unsolicited positive feedback from parents, and feel that the cultural goodwill shown by ALYN as an institution has led to a calmer environment, more open dialogue between staff and patients (and between members of staff themselves), and fewer incidents of violent behavior (both verbal and physical) than in the past. Further research quantifying the impact of improved cultural competence on patient adherence to treatment plans, frequency of medicolegal complaints, and patient satisfaction could facilitate more extensive adoption of this goal by healthcare facilities.

## Conclusions

Our experience at ALYN shows that cultural competence can be successfully adopted as an institutional value even in the cultural pressure-cooker of Jerusalem. We believe this value can and should be adopted throughout the Israeli healthcare system as a whole.

## Data Availability

Not applicable.
